# Differential alterations in the small intestine epithelial cell turnover during acute and chronic infection with *Echinostoma caproni* (Trematoda)

**DOI:** 10.1186/s13071-015-0948-5

**Published:** 2015-06-18

**Authors:** Alba Cortés, Carla Muñoz-Antoli, Carla Martín-Grau, J. Guillermo Esteban, Richard K. Grencis, Rafael Toledo

**Affiliations:** Departamento de Parasitología, Facultad de Farmacia, Universidad de Valencia, Av. Vicente Andrés Estellés s/n, 46100 Burjassot, Valencia Spain; Faculty of Life Sciences, University of Manchester, Manchester, M13 9PT UK

**Keywords:** *Echinostoma caproni*, BrdU, Intestine, Proliferation, Cell turnover, Expulsion, Chronicity

## Abstract

**Background:**

The intestinal epithelium plays a multifactorial role in mucosal defense. In this sense, augmented epithelial cell turnover appears as a potential effector mechanism for the rejection of intestinal-dwelling helminths.

**Methods:**

A BrdU pulse-chase experiment was conducted to investigate the infection-induced alterations on epithelial cell kinetics in hosts of high (mouse) and low (rat) compatibility with the intestinal trematode *Echinostoma caproni*.

**Results:**

High levels of crypt-cell proliferation and tissue hyperplasia were observed in the ileum of infected mice, coinciding with the establishment of chronic infections. In contrast, the cell migration rate was about two times higher in the ileum of infected rats compared with controls, with no changes in tissue structure, indicating that an accelerated cell turnover is associated with worm expulsion.

**Conclusion:**

Our results indicate that *E. caproni* infection induces a rapid renewal of the intestinal epithelium in the low compatible host that may impair the establishment of proper, stable host-parasite interactions, facilitating worm clearance.

## Background

Intestinal-dwelling helminths maintain an intimate contact with the intestinal epithelium, thus alterations in the intestinal architecture and/or homeostasis affect their microhabitat and can compromise the stability of parasites located at this environment. These changes are normally under the control of the immune system, which is able to manage a vast number of effector mechanisms that are directed to degrade, disable and/or dislodge intestinal helminths with the ultimate aim of inducing their expulsion from the host organism. It has been shown that immune-mediated expulsion of intestinal helminths can involve the interplay between CD4+ T cells and the gut epithelium [1–3]. Increased epithelial cell turnover is a potential dislodging mechanism that may affect the steadiness of worms in their intestinal microhabitat and, therefore, result in parasite clearance [4–8]. However, the effectiveness of this mechanism strongly depends on the host-parasite system, since they may be affected by diverse parasite evasion strategies that render the host susceptible [3]. In this context, the use of host-parasite systems that display different immune responses and susceptibility to infection arises as a useful approach to investigate the way these mechanisms can influence the course of the infection.

*Echinostoma caproni* (Trematoda: Echinostomatidae) is an intestinal trematode with no tissue phase in the definitive host. After infection, metacercariae excyst in the duodenum and the juvenile worms migrate to the posterior third of the small intestine where they attach to the intestinal mucosa through the ventral sucker [9, 10]. Although *E. caproni* is able to parasitize a wide range of laboratory rodent hosts, its compatibility differs markedly among rodent species, mainly depending on the characteristics of the immune response generated against the infection in each host species [10–12]. ICR mice are high-compatible hosts that develop chronic infections associated with strong Th1-type local immune responses. Conversely, rats act as low-compatible hosts, in which the early rejection of worms is mediated by the expansion of the local Th2 lineage, concomitantly with Th17 lineage at the expense of Th1 [13, 14]. Because of these features, the *E. caproni*-rodent model provides suitable systems to elucidate several aspects of the host-parasite relationships in intestinal infections, such as the induction of distinct effector mechanisms and their effectiveness in parasite clearance.

Although goblet cell hyperplasia and enhanced mucus production are normally considered Th2-driven effects associated with worm rejection, we have recently shown that these are not effective mechanisms to induce *E. caproni* expulsion [15]. In *E. caproni-*infected ICR mice, intestinal expression of the gel-forming mucin Muc2 is augmented at the site of infection, together with greater goblet cell-counts and an increase in both glycocalyx and mucus layer thickness. These changes coincide with the development of a Th1-type local response and the chronic establishment of the infection. In rats infected with *E. caproni*, however, the regeneration of the intestinal tissue appears to be a major effector mechanism for the early expulsion of this helminth. A differential proteomic analysis revealed that after *E. caproni* infection most of the up-regulated proteins in the rat ileal epithelial cells were related to the cytoskeleton and the maintenance of the functional integrity of the intestinal epithelium [16]. In this context, the study of infection-induced alterations on the intestinal epithelium may provide important information as regards the mechanisms determining the expulsion of worms. In the present paper, the changes induced on intestinal epithelial cell (IEC) kinetics after *E. caproni* infection are investigated in low- and high-compatible hosts to get a better understanding of the effector mechanisms involved in the rejection of *E. caproni* adult worms.

## Methods

### Parasite, hosts and experimental infections

The strain of *E. caproni*has been described previously [17]. Encysted metecercariae of *E. caproni* were removed from experimentally infected *Biomphalariaglabrata* snails and used to infect ICR male mice and albino Wistar male rats. A total of 30 ICR mice, weighing 30–35 g, and 20 rats, weighing 80–100 g, were each infected by gastric gavage with 75 and 100 metacercariae of *E. caproni*, respectively. Moreover, a total of 5 mice and 5 rats were left uninfected and used as negative controls. The end point of the experiment was set at 4 weeks post infection (wpi) in rats, since from this time, post infection, natural worm expulsion normally occurs in this host species. In mice, the experiment was extended up to the 6 wpi, as chronic infections are developed in this host. All the animals were maintained under conventional conditions with food and water *ad libitum*. This study has been approved by the Ethical Committee of Animal Welfare and Experimentation of the University of Valencia (Ref#A18348501775).

### BrdU pulse-chase analysis of the intestinal epithelial cell kinetics

A pulse-chase experiment with 5-bromo-2-deoxyuridine (BrdU) was conducted to measure the IEC migration rate along the crypt-villus axis over a defined period of time. Briefly, before infection and each 2 wpi, groups of 5 mice and 5 rats were intraperitoneally injected with 10 and 20 mg of BrdU (Sigma-Aldrich®), respectively, and were sacrificed 1 or 24 h later. Injections for the 1 h period were administered at 10.00 a.m. and, for the 24 h period, at 11.00 a.m. All mice and rats were sacrificed at 11.00 a.m. The synchronization was important to minimize any differences in turnover attributable to variations due to circadian rhythm.

Intestinal samples (0.7-1 cm length) from the site where worms were located were collected, fixed in 4 % buffered formalin and processed for paraffin embedding. Immunostaining of BrdU was performed on 4 μm paraffin sections, using a monoclonal anti-BrdU antibody (mouse anti-BrdU MoBU-1 clone, Life Technologies™) labeled with Alexa® Fluor 555dye (excitation/emission maxima: 555/580 nm). The staining protocol was established following the recommendations of Wojtowicz and Kee [18]. In brief, intestinal sections were deparaffinized in xylene for 1 h, rehydrated in alcohol and washed in PBS. DNA denaturation was performed incubating the sections in 1.5 M HCl for 30 min at 60 °C. Then, the acid was neutralized by rinsing the sections 3 times, 5 min each, in PBS. Before BrdU staining, sections were treated with NaBH_4_ 1 % to remove tissue auto-fluorescence, and blocked for unspecific unions with naïve donkey serum, 5 % in PBS containing 0.2 % Triton-X 100 (PBS-TX), for 1 h at room temperature. Thereafter, sections were rinsed 3 times in PBS, 5 min each, and incubated with anti-BrdU antibody, diluted 1/100 in PBS-TX, for 16 h at 4 °C in darkness. Finally, tissue sections were washed in PBS, as described before, and cell nuclei were counterstained with DAPI before mounting with Fluoromount (Sigma-Aldrich®) procedure.

Stained sections were examined under a fluorescent microscope (Leica DMR). Digital images were taken and the analysis was performed, blindly, by scoring 50 well-oriented villus-crypt units (VCU) per animal, 5 animals per group. Each VCU was scored for BrdU positive cells using a positional analysis. The position occupied by each BrdU-labeled cell along the crypt-villus axis was recorded, starting at the base of the crypt and finishing at the lumen. The position of labelled cells at 1 and 24 h post BrdU injection was determined, and the IEC migration rate calculated.

### Measurement of crypt and villus length

Tissue sections (4 μm thick) were stained with hematoxylin-eosin and examined under a light microscope. Representative images were taken and crypt and villus length were measured employing *ImageJ* software. For each animal, measurements were done blinded in 30 well-oriented VCU.

### Statistical analysis

Results are expressed as mean ± standard deviation. To determine time-related statistical differences in BrdU-labelled cell scoring and crypt and villus length in each host species, two-way ANOVA was conducted using the wpi and the infection status as independent variables. Bonferroni *t* test was performed as a *post hoc* analysis and differences between means were considered statistically significant when *p* < 0.05. The comparison of basal mean values between uninfected hosts (control mice *vs.* control rats) was performed by Student *t* test for unrelated samples. A value of *p* < 0.05 was considered as significant. Prior to analysis, data were log transformed to achieve normality and verified by Shapiro-Wilk test.

## Results

### Worm recovery

The weekly worm recovery from mice and rats employed in the BrdU pulse-chase experiment is shown in Table 1. In rats, worms were only recovered at 2 and 4 wpi. The number of worms collected per rat ranged from 7 to 44 (21.5 ± 13.8). In mice the experiment was extended up to the 6 wpi and the number of worms recovered per mouse in this period range from 21 to 50 (37.1 ± 11.2).

**Table 1:** *Echinostoma caproni* worm recovery from infected mice and rats intraperitoneally injected with BrdU at different weeks post infection (wpi)

Host	wpi	Worm recovery (mean ± sd)	Range
Mouse	2	37.6 ± 4.2	31–46
	4	37.2 ± 10.5	28–50
	6	36,4 ± 9,6	21–50
Rat	2	27.4 ± 10.8	16–44
	4	11.7 ± 4.5	7–16

*sd* standard deviation

### Levels of intestinal epithelial cell proliferation

**Fig. 1:**
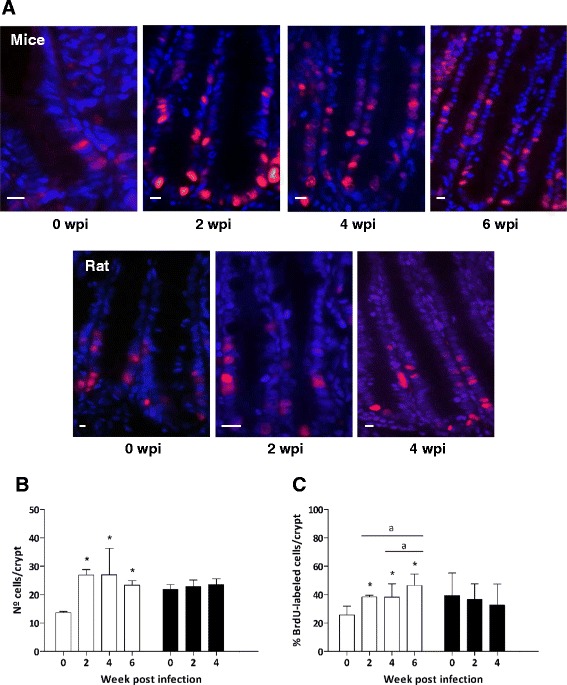
**a** Immunohistochemistry anti-BrdU on small intestine tissue sections from control and infected mice and rats 1 h after BrdU intraperitoneal administration (wpi: week post infection). Cell nuclei were stained with DAPI (blue) and BrdU-positive nuclei are pink. Scale bar: 10 μm. **b** Number of cells per crypt of Lieberkühn and **c** percentage of BrdU-labeled cells per crypt 1 h after BrdU administration in mice (white bars) and rats (black bars) at different wpi. Results are presented as mean ± standard deviation. Asterisks indicate significant differences with respect to uninfected controls (0 wpi) for each host species (*p < 0.001). Horizontal bars indicate differences between infected animals at different wpi (a: *p* < 0.05)

The levels of IEC undergoing proliferation were assessed on sections from animals necropsied 1 h post administration of BrdU. At this time point, all proliferating cells were confined in the crypts of Lieberkühn in both control and *E. caproni*-infected mice and rats (Fig. 1a). In order to provide standardized results, for each VCU analyzed, the number of BrdU-labeled cells and the number of total cells per crypt were recorded and results are presented in terms of percentage of proliferating cells per crypt.

The scoring of BrdU-labeled cells showed that in *E. caproni*-infected mice IEC proliferation levels were significantly augmented at the site of infection, and was reflected by an increase in both crypt-cell counts and percentage of proliferating cells per crypt from 2 wpi (*p* < 0.001). Moreover, these increases became greater as the course of infection progressed, reaching significantly higher values at 6 than at 2 and 4 wpi (*p* < 0.05) (Fig. 1b and c). In rats, however, 1 h after BrdU administration a slight decrease in the proliferation levels was observed in the intestine of *E. caproni*-infected animals, although, overall no significant differences were detected in the counts of BrdU-labeled cells nor the numbers of total cells per crypt in this host species (Fig. 1b and c).

Moreover, the basal proliferative rate at 0 wpi was clearly different between the two hosts (Fig. 1). Statistical comparison of the results obtained in control animals of the two species revealed that the numbers of total cells per crypt, as well as the percentages of proliferative cells, were significantly higher in control rats than in uninfected mice (*p* < 0.001). These basal interspecific differences prevented the statistical comparison of infection-induced changes between the two host species.

### Intestinal epithelial cell kinetics

**Fig. 2:**
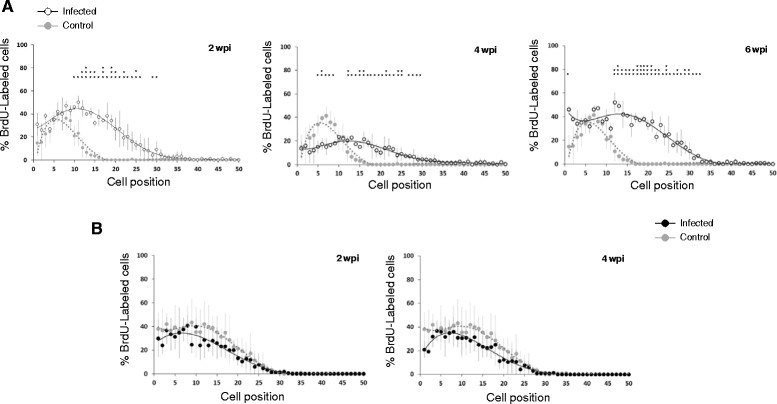
Positional distribution of BrdU-labeled cells in mice (**a**) and rats (**b**) at different weeks post *Echinostoma caproni* infection (wpi). Sections from animals killed 1 h after intraperitoneal injection were scored (50 crypts per animal, 5 animals per group) to determine the percentage of labeled cells at each position (mean ± standard deviation). Asterisks indicate significant differences with respect to uninfected controls (*p < 0.05; **p < 0.01; ***p < 0.001)

To further examine the effects of the infection on IEC kinetics, the migration rate of BrdU-labeled epithelial cells along the crypt-villus axis was compared between control and infected animals using both, 1 and 24 h post BrdU injection samples. Sections obtained 1 h after BrdU administration were employed to analyze the positional distribution of proliferative cells in the intestinal tissue. For each cell position, the percentage of times it was occupied by a BrdU-positive cell was recorded. Results for each host species at different wpi are shown in Fig. 2 (a and b). In mice, the distribution of labeled cells throughout the tissue changed after infection. The curve became displaced to the right in infected animals at each wpi studied (Fig. 2a). This change reflects not only that the number of proliferative cells was higher in the intestine of *E. caproni*-infected mice, but also that labeled cells had moved upward along the crypt axis, since higher percentages of BrdU-labeled cells were away from the crypt base in infected than in control animals. In contrast, *E. caproni*-infected rats did not show changes in the distribution of BrdU-labeled cells throughout the intestinal tissue in comparison with control animals.

**Fig. 3:**
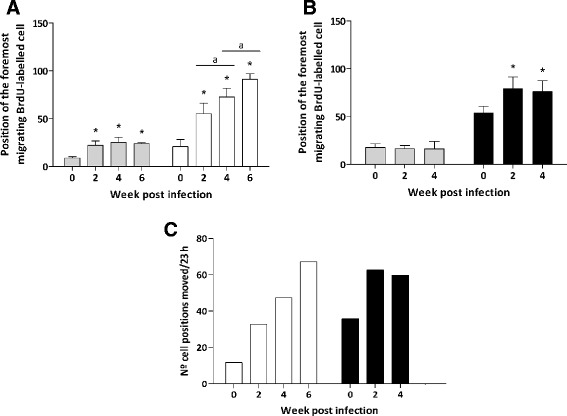
Cell position occupied by the foremost migrating BrdU-labeled cell in the crypt-villus axis in mice (**a**) and rats (**b**) after 1 h (grey bars) and 24 h (white/black bars in A and B, respectively) since the BrdU injection. Asterisks indicate significant differences with respect to uninfected controls (*p < 0.0001) for each time after BrdU administration. Horizontal bars indicate differences between infected animals at different wpi (a: *p* < 0.05). **c** The intestinal epithelial cell migration rate was assessed with a position-based analysis and expressed as the number of cell positions moved per 23 h. Mice, white bars; rats, black bars

In addition, IEC migration rate was calculated for each host at different wpi. These results showed that the position reached by the foremost migrating cell was significantly more distant from the crypt base in infected than in control animals in both host species (*p* < 0.001). In *E. caproni*-infected mice, the position reached by the foremost labeled cell was significantly further in infected than in control mice from the first hour after BrdU injection (Fig. 3a), in accordance with the results observed in the positional analysis. At this time point, the position of the leading edge of the labeled IEC cluster was similar in the three wpi studied. However, 24 h after BrdU injection the advance of labeled cells along the crypt-villus axis was significantly increased throughout the course of the infection, reaching the most distant positions at 6 wpi. The results observed in the ileum of rats were different from those described for mice. One hour after BrdU administration, the position of the last labeled cell was similar in control and infected rats (Fig. 3b). However, after 24 h the latest labeled cell was located significantly further from the crypt base in infected than in control rats. No significant differences were observed between infected animals at 2 and 4 wpi.

The results on IEC migration rate demonstrated that the advancement of cells along the crypt-villous axis occurs more rapidly in infected than in control animals of the two species. In *E. caproni*-infected mice the migration rate increased progressively along the course of the infection. Figure 3c shows that, 2 weeks after *E. caproni* metacercariae administration, the IEC migration rate was almost three-fold higher in the intestine of infected mice than in controls and it became nearly six-fold higher at 6 wpi. In the intestine of infected rats, the cell migration rate was also increased in comparison with control animals, though no differences were observed between 2 and 4 wpi. Infection-induced changes in IEC migration rates were not compared between host species due to the differences observed in basal levels (Fig. 3a and b).

### Crypt depth and villus length

**Fig. 4:**
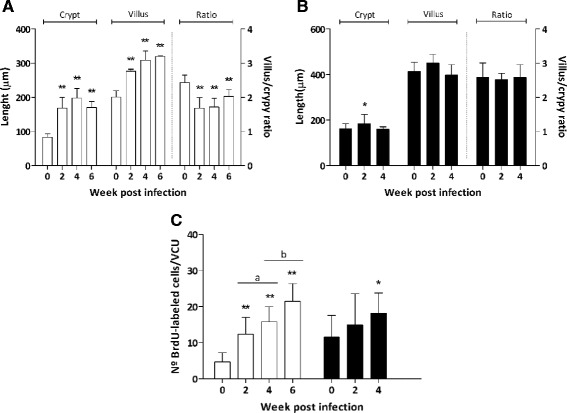
Crypt depth, villi length and villus/crypt ratio in the site of infectionwith *Echinostoma caproni* in mice (**a**) and rats (**b**) at different weeks post infection (wpi). **c** Number of BrdU-labeled cells accumulated per villus/crypt unit (VCU) 24 h after BrdU administration in the intestine of mice (white bars) and rats (black bars) at different wpi. Results are expressed as mean ± standard deviation. Asterisks indicate significant differences with respect to uninfected controls (0 wpi) for each host species (*p < 0.05; **p < 0.0001). Horizontal bars indicate differences between infected animals at different wpi (a: *p* < 0.05; b: *p* < 0.05)

BrdU pulse-chase experiment revealed that *E. caproni* infection alters proliferation levels and IEC migration rate in hosts displaying both high and low compatibility with the parasite. In order to investigate if these changes affected the intestinal tissue architecture, crypt depth and villus length were measured in the ileum of the two host species before and after intestinal infection (Fig. 4a and b).

Our study revealed that *E. caproni* infection induced a marked alteration of intestinal tissue structure in the ileum of infected mice, which was characterized by tissue hyperplasia. Crypt depth and villus length increased progressively from 2 wpi in the highly compatible host. However, the enlargement of both compartments was not proportional, resulting in an altered villus-crypt ratio, which was significantly reduced in infected mice with respect to controls (Fig. 4a). In the ileum of infected rats, in contrast, these differences were not observed. Analysis showed a slight and transient increase in crypt depth at 2 wpi (Fig. 4b), though no differences in the total number of cells per crypt were observed herein (Fig. 1b).

These results, together with those derived from BrdU labeling of proliferative cells, indicate that in highly compatible hosts the infection induces a dysregulation in the process of IEC renewal, which results into a pronounced increase in crypt and villi length. Figure 4c shows the mean number of BrdU-labeled cells counted per VCU 24 h after injection. In *E. caproni*-infected mice, proliferative cells significantly accumulated along the crypt-villus axis (*p* < 0.0001), suggesting an impaired cell turnover and causing the loss of intestinal homeostasis. In rats, tissue architecture was not affected as a consequence of the infection, although a small gain of BrdU-labeled cells per VCU was detected at 4 wpi (*p* < 0.05) (Fig. 4c).

## Discussion

Herein, we have demonstrated that *E. caproni* infection induces different IEC kinetics in the ileal mucosa of low- and high-compatible hosts. Although the presence of *E. caproni* adults induces an increase in the migration rate of IEC along the crypt-villus axis in both host species, the consequences of this change are totally different in each case, leading to a different outcome in the two hosts.

In *E. caproni*-infected mice, an elevation in the cell proliferation levels was evident from 2 wpi, coinciding with the development of tissue hyperplasia. The percentage of BrdU-labeled cells was significantly increased in the ileum of infected animals and the positional distribution of labelled cells 1 h after BrdU injection revealed that newly formed cells moved further from the crypt base as compared with controls. The analysis of positional distribution showed a similar profile at 2 and 6 wpi, though the data obtained at 4 wpi was slightly different. At this wpi, the percentage of labeled cells in positions close to the crypt base was significantly lower in infected than in control animals (cell positions 5–9). However, the importance of this is unclear, since the total number of epithelial cells and the percentage of BrdU-labeled cells per crypt were significantly higher in the ileum of infected animals with respect to uninfected controls at each wpi.

The IEC migration rate, expressed as the number of cell positions moved by one cell in 23 h, was markedly enhanced in the ileum of mice after *E. caproni* infection and increased over the course of the infection. The number of cell positions moved in this time period was almost three-fold at 2 wpi respect to uninfected controls, and it became about six-fold higher at 6 wpi. However, as it could be expected, the increase in the migration rate did not lead to an accelerated epithelial cell turnover in this host but the newly formed cells accumulated in the crypt-villus axis causing tissue hyperplasia. Although an increase in proliferation levels and cell migration rate commonly correlates with an enlarged IEC turnover, several intestinal alterations can induce the dysregulation of these processes, resulting in changes on tissue structure [19–23]. Our results indicate that *E. caproni* infection induces a dysfunction of the epithelial renewal in the ileum of mice, which is characterized by high levels of crypt-cell proliferation and a slow rate of epithelial turnover. The inverse relationship between cell proliferation rate and epithelial renewal causes the hyperplasia associated with chronicity. This is reflected by the accumulation of BrdU+ cells in the crypt-villus axis.

A critical role for IFN-γ in regulating the homeostasis of IEC proliferation during gut inflammation has been evidenced. *In vivo* blocking of this cytokine ameliorated infection-induced crypt-cell hyperproliferation in the caecum of a *Trichurismuris*-susceptible mouse strain [24]. Moreover, the IFN-γ-induced chemokine CXCL10 was also shown to reduce the rate of epithelial cell turnover in different mouse models [7, 25].

Chronic infections with *E. caproni* in mice are associated with intense local inflammatory responses and intestinal tissue damage [26, 27]. *E. caproni* infection induces strong local Th1-type responses in ICR mice, which are characterized by high levels of IFN-γ [14]. In this context, Cortés *et al.* [28] showed that local IFN-γ production in this host strain seems to play a dichotomous role, inhibiting protective Th2 responses and inducing inflammation and, concomitantly, reducing the severity of the disease and protecting ICR mice from infection-induced morbidity and mortality. INF-γ^−/−^ mice, however, exhibit an enhanced intestinal pathology and lethality after *E. caproni* infection. Intestinal homeostasis is also affected in this mouse strain, but in a different manner in comparison with *E. caproni*-infected ICR mice. In the knock out mice, the lack of IFN-γ induces crypt hyperplasia in the absence of changes in villi length, causing an altered rostral-caudal gradient that consists in an expansion of the proliferating compartment along with a non-proportional production of differentiated IEC. These differences between strains suggest that, although IFN-γ seems to play a major role in the infection-induced homeostatic dysregulation in the ileum of *E. caproni*-infected ICR mice, INF-γ-independent pathways may also be implicated. For instance, down-regulation of manganese superoxide dismutase have been shown to promote cell proliferation via activator protein 1 [29] and IEC isolated from *E. caproni*-infected mice at 2 wpi display reduced protein expression of this antioxidant enzyme (unpublished data). Moreover, the fact that increased IEC proliferation may be caused by the parasite itself through the release of mitogenic excretory/secretory products (ESP) cannot be discarded. The existence of helminth-derived factors that can influence host IEC proliferation supports this hypothesis [30–32].

The low-compatible host (rat) did not develop crypt nor villi hyperplasia as a consequence of the infection, though IEC kinetics was affected. The results obtained from the BrdU pulse-chase experiment were somewhat confusing in this host. No significant changes in the number of labeled cells were detected between control and infected animals 1 h after BrdU administration, and positional distribution analysis showed similar profiles during the complete experiment. Nevertheless, 24 h after intraperitoneal injection, differences in cell migration rate were observed between control and infected rats. These results appear to be inconsistent, since an elevation in cell migration rate does correlate with an increase in cell proliferation levels. However, it must be taken in account that intestinal epithelial renewal is subjected to the control of circadian rhythms. Although several lines of evidence suggest the existence of a crosstalk between molecules that are responsible for the generation of circadian rhythms and molecules that control cell cycle progress [33], the regulatory mechanisms behind the circadian rhythmicity of enterocyte proliferation remain elusive [34]. Several studies indicate that in rodents, the peak of the S phase of the cell cycle occurs late at night, whereas the mitotic activity is the highest in the early part of the day and the lowest around the early evening [35, 36]. BrdU is a synthetic nucleotide that is incorporated into the DNA double-chain during the replication of genetic material in the S phase of the cell cycle [37, 38]. Thus, considering that BrdU injections were carried out in the morning (10.00 a.m.), it is possible that the discrepancy between the results observed at 1 and 24 h after BrdU injection may reflect the fact that the time of highest BrdU bioavailability in the intestine did not coincide with the peak of S phase. Moreover, the magnitude of the changes detected in the ileum of infected rats was smaller than in mice, which may help to explain the absence of detectable variations during the first hour after BrdU injection in this host. The results obtained at 24 h post BrdU administration showed that IEC migration rate is about two-fold higher in infected animals than in controls. Furthermore, although the number of BrdU-labeled cells per VCU was slightly higher in the ileum of infected rats, the accumulation of newly formed epithelial cells was scarce in this host species. Altogether, these results suggest that *E. caproni* infection affects the IEC kinetics in the ileum of rats, resulting in an accelerated epithelial cell turnover associated with worm rejection.

The early expulsion of *E. caproni* adult worms is associated with the expansion of the local Th2 lineage concomitantly with the Th17 lineage at the expense of Th1 [13, 14]. Inflammation and mucosal damage are mild in this host, and the elevated levels of IL-13 expression in the intestinal tissue has been regarded as the most relevant factor determining the worm expulsion due to the alterations this cytokine is able to mediate in the intestine of the host [12, 14]. Recently, Muñoz-Antolí *et al.* [16] showed that most of the proteins overexpressed in the ileum of *E. caproni*-infected rats were related to the cytoskeleton and the maintenance of the functional integrity of the intestinal epithelium, suggesting that the regeneration of the intestinal tissue is a major effector mechanism determining the early expulsion of adult worms. Herein, we have demonstrated that intestinal epithelial renewal is accelerated after *E. caproni* infection in the rat and IL-13 may be a key immune mediator, since it has an important role in regulating the epithelial cell turnover. Cliffe *et al.* [7] found out that *T. muris* rejection was associated with an elevated rate of intestinal epithelial renewal regulated in an IL-13-dependent manner.

Cliffe *et al.* [7] proposed a new mechanism for *T. muris* expulsion in which the elevation of IEC migration rate acts as an epithelial escalator, displacing worms from their optimal niche and causing their expulsion. However, the mechanism that operates in the case of *E. caproni* infection must be different. *T. muris* larvae adults live completely or partially embedded in the host intestinal epithelium itself, thus an increased IEC turnover moves them along the crypt axis towards the caecal lumen. In contrast, *E. caproni* adult worms attach to the small intestine mucosal surface through the ventral sucker, in an active process in which they detach and attach again in close places. In this context, molecular interactions between the parasites and the IEC appear to be of relevance [39]. Therefore, an accelerated epithelial cell turnover may hinder the establishment of proper host-parasite interactions at molecular level, impairing the development of long-lasting infections. Conversely, in *E. caproni*-infected mice the slow cell turnover and tissue hyperplasia would enable more stable interactions favouring the development of chronic infections.

In summary, the results presented herein demonstrate that *E. caproni* infection induces distinct alterations in the intestinal mucosa homeostasis in hosts of high and low compatibility. The nature of these alterations appear to be related to the immune response generated in each host species and, although the precise mechanisms operating in each case are still not fully understood, these changes in the IEC kinetics may play a major role in the outcome of the infection.

## Conclusions

Alterations induced by *E. caproni* in the intestinal mucosa are dependent on the compatibility of the host. In hosts of high compatibility (mice), the infection induces high levels of crypt-cell proliferation and tissue hyperplasia, whereas in low compatible hosts (rats) the most relevant feature is an increased cell migration with no changes in tissue structure, this may be related to the early worm expulsion in rats. This suggests that an accelerated cell turnover can be an effective mechanism for the expulsion of intestinal helminths.
